# Supplementary material for: Fatigue and resilience in Master’s and PhD students in the Covid-19 pandemic in Brazil: A cross-sectional study
Source: PLoS One. 2023 Dec 1;18(12):e0295218. doi: 10.1371/journal.pone.0295218 (PMC10691712; doi:10.1371/journal.pone.0295218)
Supplement: S1 Questionnaires — (DOCX) [file pone.0295218.s002.docx]

**QUESTIONNAIRE ON SOCIODEMOGRAPHIC AND ACADEMIC DATA**

Age: _______ (years)

Sex:

- 1: Male
- 2: Female

Marital status:

- 1: With partner
- 2: Without partner

Do you have children?

- 1. No
- 2. Yes

Region of Brazil you live in**:**

- 1. North
- 2. Northeast
- 3. South
- 4. Southeast
- 5. Centre-west

State:________

What is your monthly income?

- 1. From 1 to 2 minimum salaries
- 2. From 3 to 4 minimum salaries
- 3. More than 5 minimum salaries

Do you receive additional income?

- 1. No
- 2. Yes

What is your course?

- 1. MA
- 2. PhD

In which area of knowledge?

- 1. Biological Sciences
- 2. Exact and Earth Sciences
- 3. Engineering
- 4. Health Sciences
- 5. Agrarian Science
- 6. Applied Social Sciences
- 7. Humanities Sciences
- 8. Linguistics, Letters and Arts
- 9. Multidisciplinary

Institution:

- 1. Public
- 2. Private

What is the concept for your postgraduate programme?

- 1. 1 to 3
- 2. 4 to 5
- 3. 6 to 7

Source of income:

- 1. Fixed Work
- 2. Family Resources
- 3. Scholarship
- 4. Informal Work
- 5. Unemployment Insurance
- 6. Retired
- 7. Others. Specify____________

Has the pandemic had an impact on your chronogram?

- 1. No
- 2. Yes

If yes, in which phase of the project?

- 1. Project Writing and Qualification Exam
- 2. Data Collection and Data Analysis
- 3. Final Writing
- 4. Taking the courses
- 5. Others. Specify____________

Do you consider your monthly income sufficient?

- 1. No
- 2. Yes

Has the pandemic had an impact on your income?

- 1. No
- 2. Yes

**RESILIENCE SCALE**

"Instructions" Please mark the number that indicates how much you agree or disagree with each item.

Q1: When I make plans, I carry them through.

Q1-1. I totally disagree

Q1-2.

Q1-3.

Q1-4.

Q1-5.

Q1-6.

Q1-7. I totally agree

Q2: I usually cope with problems in one way or another.

Q2-1. I totally disagree

Q2-2.

Q2-3.

Q2-4.

Q2-5.

Q2-6.

Q2-7. I totally agree

Q3: I am able to depend on myself more than anyone else.

Q3-1. I totally disagree

Q3-2.

Q3-3.

Q3-4.

Q3-5.

Q3-6.

Q3-7. I totally agree

Q4: Maintaining interest in things is important for me.

Q4-1. I totally disagree

Q4-2.

Q4-3.

Q4-4.

Q4-5.

Q4-6.

Q4-7. I totally agree

Q5: I can stay alone if necessary.

Q5-1. I totally disagree

Q5-2.

Q5-3.

Q5-4.

Q5-5.

Q5-6.

Q5-7. I totally agree

Q6: I feel proud to have accomplished things in my life.

Q6-1. I totally disagree

Q6-2.

Q6-3.

Q6-4.

Q6-5.

Q6-6.

Q6-7. I totally agree

Q7: I usually accept things without too much worry.

Q7-1. I totally disagree

Q7-2.

Q7-3.

Q7-4.

Q7-5.

Q7-6.

Q7-7. I totally agree

Q8: I am my own friend.

Q8-1. I totally disagree

Q8-2.

Q8-3.

Q8-4.

Q8-5.

Q8-6.

Q8-7. I totally agree

Q9: I feel I can deal with several things at the same time.

Q9-1. I totally disagree

Q9-2.

Q9-3.

Q9-4.

Q9-5.

Q9-6.

Q9-7. I totally agree

Q10: I’m determined.

Q10-1. I totally disagree

Q10-2.

Q10-3.

Q10-4.

Q10-5.

Q10-6.

Q10-7. I totally agree

Q11: I seldom think about the aim of things.

Q11-1. I totally disagree

Q11-2.

Q11-3.

Q11-4.

Q11-5.

Q11-6.

Q11-7. I totally agree

Q12: I do things one day at a time.

Q12-1. I totally disagree

Q12-2.

Q12-3.

Q12-4.

Q12-5.

Q12-6.

Q12-7. I totally agree

Q13: I can face difficult times because I have experienced difficulties before.

Q13-1. I totally disagree

Q13-2.

Q13-3.

Q13-4.

Q13-5.

Q13-6.

Q13-7. I totally agree

Q14: I am disciplined.

Q14-1. I totally disagree

Q14-2.

Q14-3.

Q14-4.

Q14-5.

Q14-6.

Q14-7. I totally agree

Q15: I maintain interest in things.

Q15-1. I totally disagree

Q15-2.

Q15-3.

Q15-4.

Q15-5.

Q15-6.

Q15-7. I totally agree

Q16: I usually find a motive to laugh.

Q16-1. I totally disagree

Q16-2.

Q16-3.

Q16-4.

Q16-5.

Q16-6.

Q16-7. I totally agree

Q17: My belief in myself gets me through difficult times.

Q17-1. I totally disagree

Q17-2.

Q17-3.

Q17-4.

Q17-5.

Q17-6.

Q17-7. I totally agree

Q18: In an emergency, I am a person people can count on.

Q18-1. I totally disagree

Q18-2.

Q18-3.

Q18-4.

Q18-5.

Q18-6.

Q18-7. I totally agree

Q19: I can usually look at a situation in several ways.

Q19-1. I totally disagree

Q19-2.

Q19-3.

Q19-4.

Q19-5.

Q19-6.

Q19-7. I totally agree

Q20: Sometimes I make myself do things whether I want to or not.

Q20-1. I totally disagree

Q20-2.

Q20-3.

Q20-4.

Q20-5.

Q20-6.

Q20-7. I totally agree

Q21: My life has meaning.

Q21-1. I totally disagree

Q21-2.

Q21-3.

Q21-4.

Q21-5.

Q21-6.

Q21-7. I totally agree

Q22: I don't insist on things I can't do anything about.

Q22-1. I totally disagree

Q22-2.

Q22-3.

Q22-4.

Q22-5.

Q22-6.

Q22-7. I totally agree

Q23: When I’m in a difficult situation, I normally find a way out.

Q23-1. I totally disagree

Q23-2.

Q23-3.

Q23-4.

Q23-5.

Q23-6.

Q23-7. I totally agree

Q24: I have enough energy to do what I have to do.

Q24-1. I totally disagree

Q24-2.

Q24-3.

Q24-4.

Q24-5.

Q24-6.

Q24-7. I totally agree

Q25: I don’t worry if people don’t like me.

Q25-1. I totally disagree

Q25-2.

Q25-3.

Q25-4.

Q25-5.

Q25-6.

Q25-7. I totally agree.

Piper Fatigue Scale – Revised

“Instructions”

For each question below, mark the number that best describes the fatigue you are feeling NOW. Please try to answer each question to the best of your ability. Thank you.

Q1: How long have you been feeling fatigued? (mark only one answer and complete the number of days, weeks, etc.).

Q1- 1. Days_____________

Q1- 2. Weeks__________

Q1- 3. Months____________

Q1- 4. Hours____________

Q1- 5. Minutes___________

Q2: How much stress is fatigue now making you feel?

Q2-0. None

Q2-1.

Q2-2.

Q2-3.

Q2-4.

Q2-5.

Q2-6.

Q2-7.

Q2-8.

Q2-9.

Q2-10. A lot of stress

Q3. How much does fatigue interfere with your ability to complete your work or university activities?

Q3-0. Not at all

Q3-1.

Q3-2.

Q3-3.

Q3-4.

Q3-5.

Q3-6.

Q3-7.

Q3-8.

Q3-9.

Q3-10. A lot

Q4. How much does fatigue interfere with your ability to visit or be with your friends?

Q4-0. Not at all

Q4-1.

Q4-2.

Q4-3.

Q4-4.

Q4-5.

Q4-6.

Q4-7.

Q4-8.

Q4-9.

Q4-10. A lot

Q5. How much does fatigue interfere with your ability to have sexual activity?

Q5-0. Not at all

Q5-1.

Q5-2.

Q5-3.

Q5-4.

Q5-5.

Q5-6.

Q5-7.

Q5-8.

Q5-9.

Q5-10. A lot

**Q6.** In general, how much does fatigue interfere with your ability to do any type of activity you enjoy?

Q6-0. Not at all

Q6-1.

Q6-2.

Q6-3.

Q6-4.

Q6-5.

Q6-6.

Q6-7.

Q6-8.

Q6-9.

Q6-10. A lot

Q7. How would you describe the intensity or amount of fatigue you are feeling right now?

Q7-0. Light

Q7-1.

Q7-2.

Q7-3.

Q7-4.

Q7-5.

Q7-6.

Q7-7.

Q7-8.

Q7-9.

Q7-10. Intense

Answer questions 8-12 based on the following question: (How would you describe the fatigue you are feeling right now?)

Q8. How would you describe the fatigue you are feeling right now?

Q8-0. Pleasant

Q8-1.

Q8-2.

Q8-3.

Q8-4.

Q8-5.

Q8-6.

Q8-7.

Q8-8.

Q8-9.

Q8-10. Unpleasant

Q9. How would you describe the fatigue you are feeling right now?

Q9-0. Acceptable

Q9-1.

Q9-2.

Q9-3.

Q9-4.

Q9-5.

Q9-6.

Q9-7.

Q9-8.

Q9-9.

Q9-10. Unacceptable

Q10. How would you describe the fatigue you are feeling right now?

Q10-0. Protective

Q10-1.

Q10-2.

Q10-3.

Q10-4.

Q10-5.

Q10-6.

Q10-7.

Q10-8.

Q10-9.

Q10-10. Destructive

Q11. How would you describe the fatigue you are feeling right now?

Q11-0. Positive

Q11-1.

Q11-2.

Q11-3.

Q11-4.

Q11-5.

Q11-6.

Q11-7.

Q11-8.

Q11-9.

Q11-10. Negative.

Q12. How would you describe the fatigue you are feeling now?

Q12-0. Normal.

Q12-1.

Q12-2.

Q12-3.

Q12-4.

Q12-5.

Q12-6.

Q12-7.

Q12-8.

Q12-9.

Q12-10. Abnormal.

Q13. To what extent are you feeling...

Q13-0. Strong.

Q13-1.

Q13-2.

Q13-3.

Q13-4.

Q13-5.

Q13-6.

Q13-7.

Q13-8.

Q13-9.

Q13-10. Weak.

Q14. To what extent are you feeling...

Q14-0. Awake

Q14-1.

Q14-2.

Q14-3.

Q14-4.

Q14-5.

Q14-6.

Q14-7.

Q14-8.

Q14-9.

Q14-10. Sleepy

Q15. To what extent are you feeling...

Q15-0. Full of life

Q15-1.

Q15-2.

Q15-3.

Q15-4.

Q15-5.

Q15-6.

Q15-7.

Q15-8.

Q15-9.

Q15-10. Apathetic

Q16. To what extent are you feeling...

Q16-0. Vigorous

Q16-1.

Q16-2.

Q16-3.

Q16-4.

Q16-5.

Q16-6.

Q16-7.

Q16-8.

Q16-9.

Q16-10. Tired

Q17. To what extent are you feeling...

Q17-0. Full of energy

Q17-1.

Q17-2.

Q17-3.

Q17-4.

Q17-5.

Q17-6.

Q17-7.

Q17-8.

Q17-9.

Q17-10. Lacking energy

Q18. To what extent are you feeling...

Q18-0. Patient

Q18-1.

Q18-2.

Q18-3.

Q18-4.

Q18-5.

Q18-6.

Q18-7.

Q18-8.

Q18-9.

Q18-10. Impatient

Q19. To what extent are you feeling...

Q19-0. Relaxed

Q19-1.

Q19-2.

Q19-3.

Q19-4.

Q19-5.

Q19-6.

Q19-7.

Q19-8.

Q19-9.

Q19-10. Tense

Q20. To what extent are you feeling...

Q20-0. Extremely happy

Q20-1.

Q20-2.

Q20-3.

Q20-4.

Q20-5.

Q20-6.

Q20-7.

Q20-8.

Q20-9.

Q20-10. Depressed

Q21. To what extent are you feeling...

Q21-0. Able to concentrate

Q21-1.

Q21-2.

Q21-3.

Q21-4.

Q21-5.

Q21-6.

Q21-7.

Q21-8.

Q21-9.

Q21-10. Unable to concentrate

Q22. To what extent are you feeling...

Q22-0. Able to remember

Q22-1.

Q22-2.

Q22-3.

Q22-4.

Q22-5.

Q22-6.

Q22-7.

Q22-8.

Q22-9.

Q22-10. Unable to remember

Q23. To what extent are you feeling...

Q23-0. Able to think clearly.

Q23-1.

Q23-2.

Q23-3.

Q23-4.

Q23-5.

Q23-6.

Q23-7.

Q23-8.

Q23-9.

Q23-10. Unable to think clearly.

Visual Analogue Fatigue Scale

At the moment what is the intensity of your fatigue?

| 0 | 1 | 2 | 3 | 4 | 5 | 6 | 7 | 8 | 9 | 10 |
| --- | --- | --- | --- | --- | --- | --- | --- | --- | --- | --- |
