# Supplementary material for: Fatigue and resilience in Master’s and PhD students in the Covid-19 pandemic in Brazil: A cross-sectional study
Source: PLoS One. 2023 Dec 1;18(12):e0295218. doi: 10.1371/journal.pone.0295218 (PMC10691712; doi:10.1371/journal.pone.0295218)
Supplement: Attachment — Submitted filename: PONE-D-21-28536_reviewer comments.docx [file pone.0295218.s003.docx]

The authors of this study raise and attempt to address an intriguing question – how did the pandemic affect postgraduate students? To achieve this, an impressively large scale questionnaire was disseminated to students throughout Brazil, resulting in over 3000 responses. Unfortunately, many of the conclusions drawn appear to be very shallow in nature and I worry about the lack of novelty presented in the article (e.g. it comes as little surprise that individuals with more financial support and whose research remained unimpacted were better off). Additionally, there appear to some statistical flaws and deeply concerning demographic questions being utilised, which overall detract from the study. I will detail such examples below, but I am sadly unconvinced that this article should be published. Lastly, your introduction is not sufficient with only one page provided to cover the entire area of study relating to the difficulties of post-graduate work (which is a large field in and of itself!).

To begin, I wish to raise the issue of your demographic questions. Without seeing the questionnaire itself (which is absent from the appendix), I can’t fully confirm this, but it appears that you asked participants if their race/colour was Yellow, White, Indigenous, Brown or Black. Not only is this deeply inappropriate, but you did not even raise that you were investigating race in your methodology. As you ultimately find no statistical relevance here, I found strongly recommend removing this data. As for why this is inappropriate, ‘Yellow’ is a deeply offensive term often used to combine many distinct Asian identities (e.g. Korean, Japanese, Chinese, Taiwanese etc.). Furthermore, did you only ask if the participants were ‘male’ or ‘female’? What of non-binary or trans people? As it stands, I can’t actually confirm what you asked but I suspect you may have fallen back onto old stereotypes.

Following on from these stereotypes, I worry about many of the conclusions drawn. You state ‘Being a woman often involves the accumulation of work and domestic functions’. While it is true that societal conventions often result in women undertaking more housework, I fail to see how this is related to their biological sex of gender identity. As written, it implies that the two are deeply connected, rather than an outcome of patriarchal values. Furthermore, you provide no references for this comment.

The next statement of concern was ‘Not having children can be an element that, by reducing the social interaction and emotional support brought by the mother-child relationship, can increase the chance of fatigue in women’. I am unsure why you feel that the lack of a parent-child support structure was only relevant to women? As written, are you suggesting that such a connection is less relevant to fathers? I see no reason why such a specific gender-based correlation should exist. This is compounded by an error is your statistical analysis, in which the *p* cut-off value does not appear to be corrected for the number of items utilised in your questionnaire. A Bonferroni correction requires you to divide the *p* value by the number of items in your questionnaire, which likely lowered the cut-off to <0.01 (the significance you saw for this comparison) and thereby may invalidate this analysis. Additionally, you provide no literature based justification for the use of correlation values as effect sizes. Traditionally, each statistical test has an associated effect size calculation which it appears you have chosen to not use.

Next on the list of statements that need justification is ‘Therefore, a study suggests that government officials periodically review the effectiveness of the isolation measures implemented and consider ways to make such measures more flexible in order not to compromise the health of the population [16] or consider the need for emergency aid as an aid to maintain the minimum family income’. I am surprised by the implication here that government agencies are not *already* taking this into account. As such, and this relates to my overall concern around the lack of novelty in this article, I’m not sure what this statement adds to the topic area of interest.

You have also stated that ‘Another important aspect is that biological science students may have been more resilient, as they faced challenges inherent to the benchtop researcher [19] and maintained good results in the resilience score’. I’m sorry, but I’m not sure what you mean by this. Are not all bench researchers facing challenges ‘inherent to the benchtop researcher’? How is the experience of Biological Science students unique? Surely not all of them experienced the frontline of the pandemic as you raise for the Health Science students? In fact, what are these grouped together and, indeed, why and how were any of the groups combined (i.e. why are the Exact Sciences and Earth, Engineering, and Agricultural Sciences combined into one?).

I have two last comments before I wrap up. Firstly, the lack of acknowledgements is surprising to me, as I would have at the very least expected an extension of gratitude to the students who took the time to complete the questionnaire. Secondly, the writing of this article, while generally reasonable, occasionally contained grammatical errors or signs of a lack of detailed proofreading. Some examples are below:

1) Therefore, a study suggests that government officials periodically review the effectiveness of the isolation measures … (I presume you mean - therefore, this study suggests …)
2) … such measures more flexible in order not to compromise the health of the population … (I presume you mean mental health).

Overall, without a full justification of the statistical testing undertaken, the removal of stereotypical assumptions, the presence of the actual questionnaire used AND the novelty of this work better highlighted, I unfortunately cannot recommend this work for publication.
