# Supplementary material for: Fatigue and resilience in Master’s and PhD students in the Covid-19 pandemic in Brazil: A cross-sectional study
Source: PLoS One. 2023 Dec 1;18(12):e0295218. doi: 10.1371/journal.pone.0295218 (PMC10691712; doi:10.1371/journal.pone.0295218)
Supplement: Attachment — Submitted filename: Reviewer Recommendation and Comments for Manuscript Number PONE.docx [file pone.0295218.s004.docx]

**Reviewer Recommendation and Comments for Manuscript Number PONE-D-21-28536**

Comments for authors:

Overall:

The study analyses the relationship between fatigue and resilience while controlling for academic and sociodemographic factors for postgraduate students in Brazil. The findings and the contribution could be strengthened by linking the discussion better to your data, and discussing what already happens in universities in terms of resilience training. Many programs already offer co-curricular and extra-curricular workshops and seminars on time management, mindfulness, etc. all of which I would argue might be helpful in mitigating fatigue and learning resilient behaviours. This is not considered in the current manuscript version. Also, it is not clear to me whether you found any particular factors to be the major ‘dealbreakers’ or ‘deal-makers’. You suggest financial hardships might be a significant factor, perhaps even more important in causing stress and fatigue than lack of social support. I would strongly recommend that you highlight and emphasise your main findings in the discussion. Overall, the paper would benefit from professional editing. A few expressions are unclear in what they mean or refer to. I hope further detailed comments below will be helpful in revising the paper.

Intro:

Page13line66: expression “behaviours that promote protection’ is odd, resilience is not a behaviour per se, rather a quality or attribute I would argue, what might be examples of resilient behaviours?

Method:

Consider explaining in a few words what a prospective study is.

Why is sense strict in italics?

P4l82-83: “All of the students who did not fully respond to the data collection” perhaps simplify to who did not answer all survey questions…

L89 Survey link capitalised

Discussion:

Overall, I suggest revising the structure to better orient the reader through your argument. Currently this section is somewhat disjointed. For example, the last paragraph on ‘resilience building strategies seems disconnected and not well integrated with the rest.

P15l295; change to impact OF increased levels of stress and anxiety

P16l312-313: which Brazilian study do you refer to here: “Different results from those found in a Brazilian study showed that 56% of students were doctoral students”

P17l338-340: the role of this sentence is unclear: “It is expected that younger students suffer more from fatigue and have a less resilient behaviour pattern when compared to older ones. “ Who expects that? Does your data speak to this directly? If so, you need to make the connection to your research clearer here. And who are the researchers in line 340, you or other research?

How would you explain the finding that females who were financially disadvantaged by covid-19 experienced more fatigue but students with children were more resilient. This question comes up naturally for the reader when reading the abstract. Consider explaining this contrasting finding.

P18l371: “marked devaluation” do you mean that academic activities became less of a preference for them? Who devalued such activities?

P18l375:	What do you mean by ‘intense adversity in this region’ here?

Limitations:

You state that finding other studies on this subject was difficult but you refer to several similar studies throughout.

P20l4090411, how realistic is it to conduct similar studies before pandemic, perhaps use a more nuanced language and say a general assessment of students’ resilience and fatigue would provide a good baseline or basis to work with.

Conclusion:

Review the second sentence and consider splitting in two.
