# Supplementary material for: Fatigue and resilience in Master’s and PhD students in the Covid-19 pandemic in Brazil: A cross-sectional study
Source: PLoS One. 2023 Dec 1;18(12):e0295218. doi: 10.1371/journal.pone.0295218 (PMC10691712; doi:10.1371/journal.pone.0295218)
Supplement: Attachment — Submitted filename: Response to Reviewers.docx [file pone.0295218.s005.docx]

São Paulo, 21 April 2022.

Dear Professor Pauline M. Ross:

We would like to thank you for the comments on our manuscript. We have made the proposed changes, and below we clarify the suggestions in red point by point.

Yours sincerely,

**____________________________________________________________________**

Ph.D. candidate at the Nursing School of University of São Paulo. Rua Doutor Enéas de Carvalho Aguiar, 419, CEP – 05403-000, Cerqueira Cesar, São Paulo, SP, Brazil. Telephone: 55 11 9 88481679. Corresponding author: izabel.chagas@usp.br

**Editor**

I have carefully considered the comments of both reviewers and read your manuscript. One reviewer has recommended rejection – but the grounds for this are not clear. It appears some cultural differences may have led them to make decisions based on colour and gender statements. Please read this with consideration – they were meant in good faith – but have not completely been sensitively articulated.

**We have read the considerations carefully; however, we emphasize that we were both surprised and disappointed with the way in which the questions of Reviewer 1 were made.**

Overall the result section of the manuscript needs significant improvement. You have used some interesting tools to determine fatigue and resilience. The result section needs more coherency and less fragmentation. One way to reduce this is to decrease the number of sub headings.

**We have made the suggested changes in the results section.**

Also, more literature is needed in the discussion – include literature on gender and other areas which support your findings.

**We have included more literature as a basis for the findings.**

2. In your Data Availability statement, you have not specified where the minimal data set underlying the results described in your manuscript can be found. PLOS defines a study's minimal data set as the underlying data used to reach the conclusions drawn in the manuscript and any additional data required to replicate the reported study findings in their entirety. All PLOS journals require that the minimal data set be made fully available. For more information about our data policy, please see <http://journals.plos.org/plosone/s/data-availability>.

Upon re-submitting your revised manuscript, please upload your study’s minimal underlying data set as either Supporting Information files or to a stable, public repository and include the relevant URLs, DOIs, or accession numbers within your revised cover letter. For a list of acceptable repositories, please see http://journals.plos.org/plosone/s/data-availability#loc-recommended-repositories. Any potentially identifying patient information must be fully anonymized.

**We have made the data available.**

3. We note that Figures 1 and 2 in your submission contain [map/satellite] images which may be copyrighted. All PLOS content is published under the Creative Commons Attribution License (CC BY 4.0), which means that the manuscript, images, and Supporting Information files will be freely available online, and any third party is permitted to access, download, copy, distribute, and use these materials in any way, even commercially, with proper attribution. For these reasons, we cannot publish previously copyrighted maps or satellite images created using proprietary data, such as Google software (Google Maps, Street View, and Earth). For more information, see our copyright guidelines: http://journals.plos.org/plosone/s/licenses-and-copyright.

We require you to either (1) present written permission from the copyright holder to publish these figures specifically under the CC BY 4.0 license, or (2) remove the figures from your submission:

a. You may seek permission from the original copyright holder of Figures 1 and 2 to publish the content specifically under the CC BY 4.0 license.

**We have included the source for the elaboration of the images in the footnote of the image. The software used for the elaboration was QGIS, which is a free and open source in the public domain. For the boundaries of states and countries, the IBGE base was used, source: IBGE/DGC. Continuous Cartographic Base of Brazil, 1:250.000 – BC250: 2017 version. Rio de Janeiro, 2017. Example of work published in PLOS based on IBGE: https://journals.plos.org/plosone/article/figures?id= 10.1371/journal.pone.0170278**

**Reviewer 1**

The authors of this study raise and attempt to address an intriguing question – how did the pandemic affect postgraduate students? To achieve this, an impressively large-scale questionnaire was disseminated to students throughout Brazil, resulting in over 3000 responses. Unfortunately, many of the conclusions drawn appear to be very shallow in nature and I worry about the lack of novelty presented in the article (e.g. it comes as little surprise that individuals with more financial support and whose research remained unimpacted were better off).

**The conclusions made were discussed and supported internationally. It is important to emphasize that a quality study does not necessarily need to have new information. In addition, our study advances scientific knowledge by presenting the relationship between fatigue and resilience in a population that deserves greater attention.**

Additionally, there appear to some statistical flaws and deeply concerning demographic questions being utilised, which overall detract from the study.

**We have made the necessary adjustments.**

I will detail such examples below, but I am sadly unconvinced that this article should be published. Lastly, your introduction is not sufficient with only one page provided to cover the entire area of study relating to the difficulties of post-graduate work (which is a large field in and of itself!).

**We have made additions to the Introduction.**

To begin, I wish to raise the issue of your demographic questions. Without seeing the questionnaire itself (which is absent from the appendix), I can’t fully confirm this, but it appears that you asked participants if their race/colour was Yellow, White, Indigenous, Brown or Black. Not only is this deeply inappropriate, but you did not even raise that you were investigating race in your methodology. As you ultimately find no statistical relevance here, I found strongly recommend removing this data.

**We have added the instruments used in the survey.**

**We have taken out the data on race and colour.**

As for why this is inappropriate, ‘Yellow’ is a deeply offensive term often used to combine many distinct Asian identities (e.g. Korean, Japanese, Chinese, Taiwanese etc.).

**We emphasize that in Brazilian culture, the term “yellow” is not offensive. In Brazil, the Brazilian Institute of Geography and Statistics (IBGE), the government agency responsible for carrying out a population census every decade, conducts a survey on the colour/race of Brazilians based on self-declaration, which when asked about their colour/race according to the following options: white, black, mixed race, indigenous or yellow (https://cnae.ibge.gov.br/en/component/content/article/95-7a12/7a12-vamos-know-o-brasil/ our-people/16049-color-or-race.html)**

Furthermore, did you only ask if the participants were ‘male’ or ‘female’? What of non-binary or trans people? As it stands, I can’t actually confirm what you asked but I suspect you may have fallen back onto old stereotypes**.**

**We only considered biological sex.**

Following on from these stereotypes, I worry about many of the conclusions drawn. You state ‘Being a woman often involves the accumulation of work and domestic functions’. While it is true that societal conventions often result in women undertaking more housework, I fail to see how this is related to their biological sex of gender identity. As written, it implies that the two are deeply connected, rather than an outcome of patriarchal values. Furthermore, you provide no references for this comment.

**We have rewritten the paragraph and supported it with literature on gender.**

The next statement of concern was ‘Not having children can be an element that, by reducing the social interaction and emotional support brought by the mother-child relationship, can increase the chance of fatigue in women’. I am unsure why you feel that the lack of a parent-child support structure was only relevant to women? As written, are you suggesting that such a connection is less relevant to fathers? I see no reason why such a specific gender-based correlation should exist.

**We have rewritten the analysis and taken out this paragraph.**

This is compounded by an error in your statistical analysis, in which the p cut-off value does not appear to be corrected for the number of items utilised in your questionnaire. A Bonferroni correction requires you to divide the p value by the number of items in your questionnaire, which likely lowered the cut-off to <0.01 (the significance you saw for this comparison) and thereby may invalidate this analysis. Additionally, you provide no literature-based justification for the use of correlation values as effect sizes. Traditionally, each statistical test has an associated effect size calculation which it appears you have chosen to not use.

**We have applied Bonferroni correction to adjust the values of hypothesis tests and have added a justification based on the literature for the use of effect size values.**

Next on the list of statements that need justification is ‘Therefore, a study suggests that government officials periodically review the effectiveness of the isolation measures implemented and consider ways to make such measures more flexible in order not to compromise the health of the population [16] or consider the need for emergency aid as an aid to maintain the minimum family income’. I am surprised by the implication here that government agencies are not already taking this into account. As such, and this relates to my overall concern around the lack of novelty in this article, I’m not sure what this statement adds to the topic area of interest.

**At the time of writing the manuscript, there were no standardized government measures. We have modified the paragraph.**

**As for the “lack of novelty” note, to date, we have not found any study of this magnitude which has evaluated more than 3,000 graduate students in Brazil during the pandemic. It is worth mentioning that when we carry out research, we do not necessarily find new data, which does not detract from the merit of the study.**

You have also stated that ‘Another important aspect is that biological science students may have been more resilient, as they faced challenges inherent to the benchtop researcher [19] and maintained good results in the resilience score’. I’m sorry, but I’m not sure what you mean by this. Are not all bench researchers facing challenges ‘inherent to the benchtop researcher’? How is the experience of Biological Science students unique? Surely not all of them experienced the frontline of the pandemic as you raise for the Health Science students?

**We have rewritten the paragraph.**

In fact, what are these grouped together and, indeed, why and how were any of the groups combined (i.e. why are the Exact Sciences and Earth, Engineering, and Agricultural Sciences combined into one?)

**We have regrouped according to the classification of areas of knowledge of the National Council for Scientific and Technological Development (CNPq), an entity linked to the Ministry of Science, Technology and Innovation to encourage research in Brazil. (https://www.gov.br/capes/pt-br/centrais-de-conteudo/TabelaAreasConhecimento_072012_atualizada_2017_v2.pdf).**

I have two last comments before I wrap up. Firstly, the lack of acknowledgements is surprising to me, as I would have at the very least expected an extension of gratitude to the students who took the time to complete the questionnaire.

**Participants in this study were acknowledged by the authors of the work during data collection. In the manuscript, we now thank the graduate students who participated in our research.**

Secondly, the writing of this article, while generally reasonable, occasionally contained grammatical errors or signs of a lack of detailed proofreading. Some examples are below:

1) Therefore, a study suggests that government officials periodically review the effectiveness of the isolation measures … (I presume you mean - therefore, this study suggests …)

2) … such measures more flexible in order not to compromise the health of the population … (I presume you mean mental health).

**A professional editor has made the necessary changes.**

Overall, without a full justification of the statistical testing undertaken, the removal of stereotypical assumptions, the presence of the actual questionnaire used AND the novelty of this work better highlighted, I unfortunately cannot recommend this work for publication.

**We have rewritten the complete justification of the statistical tests performed, removed the assumptions considered stereotyped, added the questionnaires, and highlighted the new elements of this work.**

**Reviewer 2**

The study analyses the relationship between fatigue and resilience while controlling for academic and sociodemographic factors for postgraduate students in Brazil. The findings and the contribution could be strengthened by linking the discussion better to your data, and discussing what already happens in universities in terms of resilience training. Many programs already offer co-curricular and extra-curricular workshops and seminars on time management, mindfulness, etc. all of which I would argue might be helpful in mitigating fatigue and learning resilient behaviours. This is not considered in the current manuscript version. Also, it is not clear to me whether you found any particular factors to be the major ‘dealbreakers’ or ‘deal-makers’. You suggest financial hardships might be a significant factor, perhaps even more important in causing stress and fatigue than lack of social support. I would strongly recommend that you highlight and emphasise your main findings in the discussion. Overall, the paper would benefit from professional editing. A few expressions are unclear in what they mean or refer to. I hope further detailed comments below will be helpful in revising the paper.

**We have restructured the discussion and have been helped by a professional editor.**

**Intro:**

Page13 line 66: expression “behaviours that promote protection’ is odd, resilience is not a behaviour per se, rather a quality or attribute I would argue, what might be examples of resilient behaviours?

**We have changed the paragraph and aligned it with the definition of resilience used by Wagnild and Young (Wagnild GM, Young HM. Development and psychometric evaluation of resilience scale. J Nurs Meas. 1993;1(2):165-78.)**

**Method:**

Consider explaining in a few words what a prospective study is.

**We have rewritten the paragraph/sentence.**

Why is sense strict in italics?

**A formatting error has been corrected.**

P4l82-83: “All of the students who did not fully respond to the data collection” perhaps simplify to who did not answer all survey questions…

**We have made the suggested change.**

L89 Survey link capitalised.

**We have rewritten the paragraph/sentence.**

**Limitations:**

You state that finding other studies on this subject was difficult but you refer to several similar studies throughout.

P20l4090411, how realistic is it to conduct similar studies before pandemic, perhaps use a more nuanced language and say a general assessment of students’ resilience and fatigue would provide a good baseline or basis to work with.

**We agree with the point and have rewritten the paragraph in the limitations of the study.**

Conclusion:

Review the second sentence and consider splitting it into two.

**We have rewritten the paragraph/sentence as suggested.**
