# Supplementary material for: Fatigue and resilience in Master’s and PhD students in the Covid-19 pandemic in Brazil: A cross-sectional study
Source: PLoS One. 2023 Dec 1;18(12):e0295218. doi: 10.1371/journal.pone.0295218 (PMC10691712; doi:10.1371/journal.pone.0295218)
Supplement: Attachment — Submitted filename: R2 comments.docx [file pone.0295218.s006.docx]

Thank you for revising the manuscript. I still have concerns about the suitability of this paper for publication. Reading Reviewer 1 comments and the authors’ responses further support my concerns. I will leave it to Reviewer 1 to check the technical aspects of the analysis and how the authors responded. I also echo Reviewer 1 concern about the statements made in regard to ‘yellow’. I do not think that this is appropriate for an international audience and might be highly offensive to international readers, although I take the authors’ response on board about the use of this term by Brazilian government agencies. The authors are also well advised to provide a more reflective and considerate response in regard to traditional gender roles, as per Reviewer 1 suggestions.

Please also note that my original comments on the Discussion section were not at all addressed in the authors’ response.

I have re-read the Discussion again and have further points for authors to consider:

The writing still needs a major revision throughout the manuscript, perhaps with the help of a Native English speaker? Some examples include:

‘food products’ e.g. ‘food’?

‘bringing higher levels of fatigue’ e.g. ‘causing’?

‘Overcoming the “publish or perish” mentality’, is an odd expression

“an Indian study [69] with the same theme”, a study in India? Do you mean research questions when you say ‘theme’?

“Graduate students who carry out their studies at private universities in Brazil may feel more institutionally helpless because they have a restricted involvement within the academic community in the pandemic.” Unclear and needs explaining.

This paragraph does not make clear what data it draws on:

“The northern region of Brazil was initially the most affected by the pandemic [67]. In addition, it receives less help and is the region with most economic, political, social adversities, which can influence the performance of students’ resilience levels as they have encountered a period of intense adversity in this region. Similar data have been found in northern Italy [66].” If this is not backed up by the authors’ data, why is it relevant in the discussion?

There are other similar expressions that I find difficult to understand.

The main problem I see in the discussion is that a large section of it reads like an extension of a literature review. Surprisingly little reflection and reference is given to the authors’ data, considering the significance of the sample size. I would expect much more comparing and contrasting in reference to the authors’ data segments, e.g. gender differences, academic vs low SES demographic comparison, disciplinary comparison, perhaps institutional comparison. I agree with Reviewer 1 that much deeper insights should be gained from such rich data. Unfortunately, the analysis, at least in the way it’s presented in the discussion, remains fairly superficial and doesn’t know clearly add novel insights.

The “limitations and strong points” section is weak and seems unnecessary. You could add more value by proposing a number of programs, workshops or offerings universities could add to support PhD students’ resilience building.
