# Supplementary material for: Fatigue and resilience in Master’s and PhD students in the Covid-19 pandemic in Brazil: A cross-sectional study
Source: PLoS One. 2023 Dec 1;18(12):e0295218. doi: 10.1371/journal.pone.0295218 (PMC10691712; doi:10.1371/journal.pone.0295218)
Supplement: Attachment — Submitted filename: RESPONSE TO REVIEWER.docx [file pone.0295218.s007.docx]

**RESPONSE TO REVIEWER**

**São Paulo, February 28, 2023.**

Dear Professor Pauline M. Ross:

We would like to thank you for the comments on our manuscript. We have made

the proposed changes, and below we clarify the suggestions in red point by point.

Yours sincerely,

_________________________________________________________________

Ph.D. candidate at the Nursing School of University of São Paulo. Rua Doutor

Enéas de Carvalho Aguiar, 419, CEP – 05403-000, Cerqueira Cesar, São Paulo, SP,

Brazil. Telephone: 55 11 9 88481679. Corresponding author: izabel.chagas@usp.br

**Academic Editor**

Could you please make the changes as required by the reviewer so that this review can be completed.  I notice the reviewer comments that there are sections which still are not logical in flow.  This is very important to fix in this version.  Following the revision, I will complete what I hope is a final read and edit – so that this manuscript can be resolved.  This very much depends however on the quality of the next revised version.  I encourage you to persist and perhaps obtain some assistance to produce a quality manuscript.  Best wishes and I look forward to the resolution.

**Response: We have made substantial changes to the manuscript in response to reviewers' requests and presented an improved version.**

**Reviewer 2:**

Thank you for revising the manuscript. I still have concerns about the suitability of this paper for publication. Reading Reviewer 1 comments and the authors’ responses further support my concerns. I will leave it to Reviewer 1 to check the technical aspects of the analysis and how the authors responded. I also echo Reviewer 1 concern about the statements made in regard to ‘yellow’. I do not think that this is appropriate for an international audience and might be highly offensive to international readers, although I take the authors’ response on board about the use of this term by Brazilian government agencies.

**Response: Thank you for the comments. We believe that the manuscript has publication potential as we could not find in the literature an extensive assessment of fatigue and resilience among graduate students in such a challenging scenario. We considered the suggestions of Reviewer 1 in the previous round, and, as you can see in the manuscript, the race/yellow color variable has been removed.**

The authors are also well advised to provide a more reflective and considerate response in regard to traditional gender roles, as per Reviewer 1 suggestions.

**Response: We have reconsidered traditional gender roles and our results. We have reformulated the text in the Discussion.**

Please also note that my original comments on the Discussion section were not at all addressed in the authors’ response.

**Response: We apologize for not addressing your previous points in the response letter and only changing them throughout the manuscript. In this round we address the different points.**

I have re-read the Discussion again and have further points for authors to consider: The writing still needs a major revision throughout the manuscript, perhaps with the help of a Native English speaker? Some examples include:

‘food products’ e.g. ‘food’?

‘bringing higher levels of fatigue’ e.g. ‘causing’?

‘Overcoming the “publish or perish” mentality’, is an odd expression

“an Indian study [69] with the same theme”, a study in India? Do you mean research questions when you say ‘theme’?

**Response: We have revised the text with the help of a native speaker.**

“Graduate students who carry out their studies at private universities in Brazil may feel more institutionally helpless because they have a restricted involvement within the academic community in the pandemic.” Unclear and needs explaining.

**Response: We have reformulated to Discussion text and presented an explanation for this point.**

This paragraph does not make clear what data it draws on:

“The northern region of Brazil was initially the most affected by the pandemic [67]. In addition, it receives less help and is the region with most economic, political, social adversities, which can influence the performance of students’ resilience levels as they have encountered a period of intense adversity in this region. Similar data have been found in northern Italy [66].” If this is not backed up by the authors’ data, why is it relevant in the discussion?

**Response: We have reformulated the Discussion and emphasized the main results.**

There are other similar expressions that I find difficult to understand. (Existem outras expressões semelhantes que acho difícil de entender).

**Response: We have revised the translation of the text with the help of a native speaker.**

The main problem I see in the discussion is that a large section of it reads like an extension of a literature review. Surprisingly little reflection and reference is given to the authors’ data, considering the significance of the sample size. I would expect much more comparing and contrasting in reference to the authors’ data segments, e.g. gender differences, academic vs low SES demographic comparison, disciplinary comparison, perhaps institutional comparison. I agree with Reviewer 1 that much deeper insights should be gained from such rich data. Unfortunately, the analysis, at least in the way it’s presented in the discussion, remains fairly superficial and doesn’t know clearly add novel insights.

**Response: We have reformulated the entire text of the Discussion and presented insights and considerations on the main findings. We have responded to your suggestion and have presented comparisons between gender differences, academic characteristics versus low socioeconomic status, and disciplinary and institutional differences.**

The “limitations and strong points” section is weak and seems unnecessary. You could add more value by proposing a number of programs, workshops or offerings universities could add to support PhD students’ resilience building.

**Response: We have removed the limitations and strengths section and included suggestions for programs and strategies for universities in the Discussion.**

**Reviewer 3:**

This study provides a large cross-sectional study to assess the impact of the COVID-19 pandemic on postgraduate students in Brazil. The authors should be commended in collecting and analysing a large body of data that provides a snapshot of some key instruments for analysis to assess the impact of fatigue and resilience. I can see from the response to the reviewers that the authors have made a considerable effort to review and adjust based on the feedback provided. Having not read the original submission, I can only comment directly on the revision that has been submitted.

**Response: Thank you for your comments and time spent reading this version.**

Overall, the manuscript has had much more description and link to previous studies, which does enhance the study, however, in places, the information does lack coherence and flow. There seem to be some paragraphs that are quite short (see first paragraph of introduction) that could be linked better throughout. The 7th paragraph of the introduction, the final sentence needs re-wording – “Literature data” is not correct to refer to and you should include what the significant effects were (greater/improved?).

**Response: We have reformulated the paragraphs of the introduction and revised the entire text, aiming to give it greater coherence and flow. We have readjusted the seventh paragraph of the Introduction.**

Your overall aim, in addition to analysing the level of fatigue and resilience, is to also consider the association with sociodemographic and academic factors, which I feel is what is lacking in the discussion. You do a good job of discussing the literature around fatigue and resilience, however, given you present two key figures associated with demographic data, I would like to see more detail around this. The novelty of your paper is that you are assessing Brazilian students – the demographics play a significant role here and this is the novelty in my opinion. This will allow you to utilise the nice studies you have to support your findings in other countries.

**Response: We have rewritten the entire text of the Discussion and have presented insights and thoughts on the main findings. We have included considerations on sociodemographic and academic factors.**

Whilst fatigue is not my field, I am curious as to why both a PFS and a VAFS scale are used? I may be misinterpreting this, but they seem to give slightly differing results? You also only seem to primarily discuss the PFS results. I also was unclear (for VAFS) what best result versus worst result meant? If these are common terms for these scales, please ignore, if not, please edit what this means.

**Response: We have included a justification in the Methods section “We have chosen these two instruments (PFS and VAFS) to assess fatigue, seeking to ensure a more global and reliable analysis”. The use of more than one instrument to assess the same phenomenon is not uncommon in the literature. The results of the two scales were very close to the PFS, with an average level of 6.08, and the VAFS with an average level of 6.10. What was slightly different was the inferential analysis in which the PFS seemed to be more sensitive to the characteristics that may be associated with fatigue (Source of income). We have modified the description of the result for VAFS.**

In 2.3 you refer to n number as (n), however in each table it is referred to as N – please edit.

**Response: The alteration has been made.**

Paragraph prior to table 2 starts with “The students” – I assume you mean Most students? Or “The survey found that…”.

**Response: The alteration has been made.**

The figure legends for the 2 images are underwritten. Whilst it is good you have included the source, you should also provide the reader with a description of what the images represent. For example, darker colour means more fatigue etc. I also recommend that figure 1 be given an A and B for each image to help the reader.

**Response: We have included an explanatory note for each figure and have identified the fatigue figures.**

The discussion is generally good (see comments above), but I do feel it lacks flow. I am also slightly confused with paragraph 2, where you refer to females having family commitments may have significant affects on their careers, however in your study the majority of respondents were female and did not have children? Please review this data in relation to your outcomes. It would also be interesting to see what % of the female respondents did have children.

**Response: We have reviewed the data and presented the % of women who had children. We have rewritten the Discussion and improved the analysis of gender differences.**

Similar to a previous reviewer, there is mention of many measures currently in place – I suggest you restructure this paragraph to be towards the latter section of the discussion in order to link this in with what you are also suggesting is implemented across the country. For your age data, you mention this differs from the current literature – do you have a suggestion as to why your data may differ?

**Response: We have restructured the Discussion as suggested and left the strategies and measures at the end of the section. We have added an insight into why our age data diverged from a pre-pandemic study “****We think that in the pandemic young students had fewer of their own and/or institutional resources to deal with psychic stressors. The lack of a support network and social interactions for many young people may have been risk factors for fatigue”.**

4.1 – I’m not sure you require the strong points section – this should be the focus of the discussion and come through to the reader without having to state it.
 Overall, whilst the language has been improved, I would suggest further proof reading and structural/grammar checks prior to resubmission. Thank you for your interesting study and I wish you well with the revision.

**Response: We have removed the limitations and strengths section and included it in the Discussion. We have revised the translation with a native speaker.**
