# Supplementary material for: Fatigue and resilience in Master’s and PhD students in the Covid-19 pandemic in Brazil: A cross-sectional study
Source: PLoS One. 2023 Dec 1;18(12):e0295218. doi: 10.1371/journal.pone.0295218 (PMC10691712; doi:10.1371/journal.pone.0295218)
Supplement: Attachment — Submitted filename: PLOSReviewdasChagasValota.docx [file pone.0295218.s008.docx]

**PONE-D-21-28536
"Fatigue and resilience in Master's and PhD students in the Covid-19 pandemic in Brazil: a cross-sectional study"**

When this manuscript was submitted – it was sent out for review – because it was so novel and original. The review has now been a long process, with over four reviewers at different times. One reviewer suggested a rejection, two suggested a major review and one a minor review. Each time I have suggested a major review – in light of the reviewer’s comments. Each time the revised version has improved, but not significantly enough to allow a change to a minor revision or preferably an acceptance. On each occasion because of the continued novelty and timeliness of the manuscript I have also tried to provide feedback which would be helpful to try and seek an improvement in the manuscript so that it can move towards being published.

I have decided with this final version to do the review myself – in an effort to provide the detail which I hope you may be able to use to bring this manuscript to a conclusion.

As indicated in my last set of comments – this manuscript needed a rigorous review

These are my comments from the previous review:

Could you please make the changes as required by the reviewer so that this review can be completed. I notice the reviewer comments that there are sections which still are not logical in flow. This is very important to fix in this version. Following the revision, I will complete what I hope is a final read and edit – so that this manuscript can be resolved. This very much depends however on the quality of the next revised version. I encourage you to persist and perhaps obtain some assistance to produce a quality manuscript. Best wishes and I look forward to the resolution.

This was the response: We have made substantial changes to the manuscript in response to reviewers' requests and presented an improved version.

Unfortunately although the response from the authors was positive, this 3^rd^ review has not gone far enough. There remains several coherency and language issues – which I have detailed below. There are still some interpretation issues – and general formatting of tables and figures which require further attention.

After considerable reflection, I have made the decision to allow one more major revision. If the authors decide to review again – then please seek English language assistance in the next review of the manuscript. If the manuscript does not improve considerably in language and formatting – then I will need to reject the next version. I really don’t want to reject this manuscript, given the considerable time it has taken to review – and the efforts of the authors. I urge you to make sure the next version – has dealt with the issues described here and in previous reviews. I start with the reviewers comments which I do not believe have been adequately addressed and move onto more specific comments from this version of the manuscript.

Not yet adequately addressed:

1. I am also slightly confused with paragraph 2, where you refer to females having family commitments may have significant affects on their careers, however in your study the majority of respondents were female and did not have children? Please review this data in relation to your outcomes. It would also be interesting to see what % of the female respondents did have children.
2. I have re-read the Discussion again and have further points for authors to consider: The writing still needs a major revision throughout the manuscript, perhaps with the help of a Native English speaker?
3. The main problem I see in the discussion is that a large section of it reads like an extension of a literature review. Surprisingly little reflection and reference is given to the authors’ data, considering the significance of the sample size. I would expect much more comparing and contrasting in reference to the authors’ data segments,

Detailed feedback

Introduction

Lines 75-76 Delete from “before and in…. - up until financial” in line 76. This should then read – “marked by psychological pressure with instability” and insert “with”

Line 77 replace “to” with “in”, delete “their teaching and research activities” and replace with “guidance and financial instability”.

Line 79 insert “and” between “experiments interrupt”

Line 83 delete “can lead” and replace with “have been found to”

Line 87 Replace “a” with “for example, a”

Line 88 Make “experiences” experience”

Line 89 Insert “Also” before “In”

Line 92 Delete “Given this scenario” and start at “Fatigue”

Line 113 Delete “data” and replace with “findings”

Line 120 Delete “the” and replace with “being that”

Line 121 Delete “that” and “and” and replace “Authors” with “Studies”

Line 122 Replace “state” with “have found” and delete from “has…… up until adaptive capacity” and replace with “and” and then replace “and” with “are”

Line 123 Replace “helping” with “required” and “seen as a” with “is also”

Line 124 Delete “multidimenionsal construct” and “by” and “among different elements” so that this reads “defined as the interaction between the individual and”

Line 125 delete “contains such as”

Line 126 Insert “also” in between can affect”

Line 138 Delete “probably”

Lines 133-134 Delete “and to verify” and replaced with “and to determine”

2.1 Population and recruitment.

Lines 140 Delete Stricto sensu – as this needs translation – and instead use “only those” and delete from “all areas of knowledge” – as the meaning is unclear. Move up “over 18 years of age from line 141 and put after “graduate students” and insert “who” “and” so that this reads “only those graduate students who were 18 years and over and enrolled in Brazilian educational institutions and responded to all survey items were included in this study”.

Line 142 insert “in the survey and delete “the research”

Line 145 Replace “were” with “was”

Line 147 Delete “subsequently” and “the”

Line 152 Delete “constructed by the authors” and start at “This” and add “collected sociodemographic and academic data”

Line 156 Delete “the phase of greatest impact” as meaning is unclear

Line 161 replace “assess” with “assessed” – past tense

Line 167 Move “as well as total score” to later in the text.

Line 169 Replace “is” with “was – once again should be past tense

Line 184 Delete “addresses resilience and is” and replace with “was” and on line 185 insert “resilience and “ before “levels”

3. Results

Line 224 replace “forms” with “survey”

Line 266 replace “having” with “had” – past tense required. Insert :”were” before “without”

Line 242 replace “from” with “enrolled”

4. Discussion

This need a complete reflective re-write as indicated several times by reviewers. Comments below are for English language – but I have not completed the entire re-write of this discussion – as this is up to the authors – and goes beyond what is reasonable.

Line 357 Replace “assesses” with “assessed

Line 369 Delete “students with difficulties in the two impacted stages showing”

Line 317-377 needs a complete re-write. Start with “delete “in the literature and we found that indicate the” and replace with “Other studies have found a”

Line 372 Replace “investigations” with “studies” to be consistent with previous sentence

Line 373 Insert after “fatigued” “ prior to the COVID-19 pandemic” and delete “this”

Line 374 Delete “this challenging scenario” and replace with “during the COVID-19 pandemic”

Line 374 Move up from line 377 “similar to our results” and place after “hand,”

Line 385 Delete “also” and change ”work” to “worked”

Line 390. Add full stop after [44} and start with “such change created “ and delete “bringing” and delete “the development or an” and replace with “on top of already”

Paragraph from lines 393- 401 requires a complete re-write. The changes are so many thay it is easier for me and perhaps clearer for the authors to understand – if I just write out what should be there.

So replace lines 393-406 with the following text:

In public universities in Brazil there is a constant demand for academics or researchers to increase their scientific production as they are under pressure from government classification processes which rank institutions and graduate programs on their research productivity. Researchers or academics must then prioritise their work into teaching, research and extension activities. If they are to be promoted and be competitive for jobs then they must produce publications and have success in research funding. This pressure and bureaucratic structure can cause an overload on academics which is transferred to and has a direct impact on graduate students

This is in contrast to private universities in Brazil where there is less demand for research publications and a greater focus on teaching. Academics are often on more balanced workload allocations with contracts which are not solely based on research. Graduates students also are in better financial situations and this is correlated with resilience.

It is really beyond my role to rewrite the rest of the text from lines 405-466. This needs however a complete re-write following what I have written above. I have not re-written pages 23-25 lines 426-486 – need careful re-write to bring out the main points.

However – a brief look

Line 425 – delete the entire line as this does not make sense.

Line 437 – more issues with past tense – this should be “were” delete “are”

5. Conclusion

Lines 481-486 Re write from lines 481-487.

Final comments to authors
