# Supplementary material for: Fatigue and resilience in Master’s and PhD students in the Covid-19 pandemic in Brazil: A cross-sectional study
Source: PLoS One. 2023 Dec 1;18(12):e0295218. doi: 10.1371/journal.pone.0295218 (PMC10691712; doi:10.1371/journal.pone.0295218)
Supplement: Attachment — Submitted filename: RESPONSE TO REVIEWERS.docx [file pone.0295218.s009.docx]

**RESPONSE TO REVIEWERS**

**São Paulo, August 31, 2023.**

Dear Professor Pauline M. Ross:

We would like to thank you for the comments on our manuscript. We have made the proposed changes, and below we clarify the suggestions in red point by point.

Yours sincerely,

_________________________________________________________________

Ph.D. candidate at the Nursing School of University of São Paulo. Rua Doutor

Enéas de Carvalho Aguiar, 419, CEP – 05403-000, Cerqueira Cesar, São Paulo, SP,

Brazil. Telephone: 55 11 9 88481679. Corresponding author: izabel.chagas@usp.br

**Academic Editor**

When this manuscript was submitted – it was sent out for review – because it was so novel and original. The review has now been a long process, with over four reviewers at different times. One reviewer suggested a rejection, two suggested a major review and one a minor review. Each time I have suggested a major review – in light of the reviewer’s comments. Each time the revised version has improved, but not significantly enough to allow a change to a minor revision or preferably an acceptance. On each occasion because of the continued novelty and timeliness of the manuscript I have also tried to provide feedback which would be helpful to try and seek an improvement in the manuscript so that it can move towards being published.

I have decided with this final version to do the review myself – in an effort to provide the detail which I hope you may be able to use to bring this manuscript to a conclusion.

As indicated in my last set of comments – this manuscript needed a rigorous review.

**Response: From the outset, we have tried to meet all the requests from all the reviewers. I believe that some questions have been difficult due to a lack of understanding of the request, which may be due to the difference in language and also the way in which it is written. In this version we have made every effort to meet the editor with very pertinent suggestions.**

Unfortunately although the response from the authors was positive, this 3rd review has not gone far enough. There remains several coherency and language issues – which I have detailed below. There are still some interpretation issues – and general formatting of tables and figures which require further attention.

After considerable reflection, I have made the decision to allow one more major revision. If the authors decide to review again – then please seek English language assistance in the next review of the manuscript. If the manuscript does not improve considerably in language and formatting – then I will need to reject the next version. I really don’t want to reject this manuscript, given the considerable time it has taken to review – and the efforts of the authors. I urge you to make sure the next version – has dealt with the issues described here and in previous reviews. I start with the reviewers comments which I do not believe have been adequately addressed and move onto more specific comments from this version of the manuscript.

**Response: Thank you for this opportunity to review the manuscript and for your comments on any changes.**

Reviewer comments which have not yet adequately addressed:

1. I am also slightly confused with paragraph 2, where you refer to females having family commitments may have significant affects on their careers, however in your study the majority of respondents were female and did not have children? Please review this data in relation to your outcomes. It would also be interesting to see what % of the female respondents did have children.

**Response: We have reviewed the data and presented the % of women who had children. The change is in the lines: 216-217. We have changed the discussion and emphasized the finding based on our data. The change is in the lines: 407-416.**

I have re-read the Discussion again and have further points for authors to consider: The writing still needs a major revision throughout the manuscript, perhaps with the help of a Native English speaker?

**Response: We have revised the translation of the text with the help of a native speaker.**

The main problem I see in the discussion is that a large section of it reads like an extension of a literature review. Surprisingly little reflection and reference is given to the authors’ data, considering the significance of the sample size. I would expect much more comparing and contrasting in reference to the authors’ data segments.

**Response: We have amended the discussion based on the suggestions and the suggested model paragraph. /Alteramos a discussão com base nas sugestões e no modelo de parágrafo que foi sugerido.**

Detailed feedback:

Introduction

Lines 75-76 Delete from “before and in…. - up until financial” in line 76. This should then read – “marked by psychological pressure with instability” and insert “with”.

**Response: The alteration has been made.**

Line 77 replace “to” with “in”, delete “their teaching and research activities” and replace with “guidance and financial instability”.

**Response: The alteration has been made.**

Line 79 insert “and” between “experiments interrupt”.

**Response: The alteration has been made.**

Line 83 delete “can lead” and replace with “have been found to”

**Response: The alteration has been made.**

Line 87 Replace “a” with “for example, a”

**Response: The alteration has been made.**

Line 88 Make “experiences” experience”

**Response: The alteration has been made.**

Line 89 Insert “Also” before “In”

**Response: The alteration has been made.**

Line 92 Delete “Given this scenario” and start at “Fatigue”

**Response: The alteration has been made.**

Line 113 Delete “data” and replace with “findings”

**Response: The alteration has been made.**

Line 120 Delete “the” and replace with “being that”

**Response: The alteration has been made.**

Line 121 Delete “that” and “and” and replace “Authors” with “Studies”

**Response: The alteration has been made.**

Line 122 Replace “state” with “have found” and delete from “has…… up until adaptive capacity” and replace with “and” and then replace “and” with “are”

**Response: The alteration has been made.**

Line 123 Replace “helping” with “required” and “seen as a” with “is also”

**Response: The alteration has been made.**

Line 124 Delete “multidimenionsal construct” and “by” and “among different elements” so that this reads “defined as the interaction between the individual and”

**Response: The alteration has been made.**

Line 125 delete “contains such as”

**Response: The alteration has been made.**

Line 126 Insert “also” in between can affect”

**Response: The alteration has been made.**

Line 138 Delete “probably”

**Response: The alteration has been made.**

Lines 133-134 Delete “and to verify” and replaced with “and to determine”

**Response: The alteration has been made.**

2.1 Population and recruitment.

Lines 140 Delete Stricto sensu – as this needs translation – and instead use “only those” and delete from “all areas of knowledge” – as the meaning is unclear. Move up “over 18 years of age from line 141 and put after “graduate students” and insert “who” “and” so that this reads “only those graduate students who were 18 years and over and enrolled in Brazilian educational institutions and responded to all survey items were included in this study”.

**Response: The alteration has been made.**

Line 142 insert “in the survey and delete “the research”

**Response: The alteration has been made.**

Line 145 Replace “were” with “was”

**Response: The alteration has been made.**

Line 147 Delete “subsequently” and “the”

**Response: The alteration has been made.**

Line 152 Delete “constructed by the authors” and start at “This” and add “collected sociodemographic and academic data”

**Response: The alteration has been made.**

Line 156 Delete “the phase of greatest impact” as meaning is nuclear

**Response: The alteration has been made.**

Line 161 replace “assess” with “assessed” – past tense

**Response: The alteration has been made.**

Line 167 Move “as well as total score” to later in the text.

**Response: The alteration has been made.**

Line 169 Replace “is” with “was – once again should be past tense

**Response: The alteration has been made.**

Line 184 Delete “addresses resilience and is” and replace with “was” and on line 185 insert “resilience and “ before “levels”
**Response: The alteration has been made.**

3. Results
Line 224 replace “forms” with “survey”

**Response: The alteration has been made.**

Line 266 replace “having” with “had” – past tense required. Insert :”were” before “without”

**Response: The alteration has been made.**

Line 242 replace “from” with “enrolled”

**Response: The alteration has been made.**

4. Discussion
This need a complete reflective re-write as indicated several times by reviewers.

**Response: We changed it and tried to write more reflectively.**

Comments below are for English language – but I have not completed the entire re-write of this discussion – as this is up to the authors – and goes beyond what is reasonable.
Line 357 Replace “assesses” with “assessed

**Response: The alteration has been made.**

Line 369 Delete “students with difficulties in the two impacted stages showing”

**Response: The alteration has been made.**

Line 317-377 needs a complete re-write. Start with “delete “in the literature and we found that indicate the” and replace with “Other studies have found a”

**Response: The alteration has been made.**

Line 372 Replace “investigations” with “studies” to be consistent with previous sentence

**Response: The alteration has been made.**

Line 373 Insert after “fatigued” “ prior to the COVID-19 pandemic” and delete “this” **Response: The alteration has been made.**

Line 374 Delete “this challenging scenario” and replace with “during the COVID-19 pandemic”

**Response: The alteration has been made.**

Line 374 Move up from line 377 “similar to our results” and place after “hand,”

**Response: The alteration has been made.**

Line 385 Delete “also” and change ”work” to “worked”

**Response: The alteration has been made.**

Line 390. Add full stop after [44} and start with “such change created “ and delete “bringing” and delete “the development or an” and replace with “on top of already” **Response: The alteration has been made.**

Paragraph from lines 393- 401 requires a complete re-write. The changes are so many thay it is easier for me and perhaps clearer for the authors to understand – if I just write out what should be there.
So replace lines 393-406 with the following text:
In public universities in Brazil there is a constant demand for academics or researchers to increase their scientific production as they are under pressure from government classification processes which rank institutions and graduate programs on their research productivity. Researchers or academics must then prioritise their work into teaching, research and extension activities. If they are to be promoted and be competitive for jobs then they must produce publications and have success in research funding. This pressure and bureaucratic structure can cause an overload on academics which is transferred to and has a direct impact on graduate students
This is in contrast to private universities in Brazil where there is less demand for research publications and a greater focus on teaching. Academics are often on more balanced workload allocations with contracts which are not solely based on research. Graduates students also are in better financial situations and this is correlated with resilience.

**Response: We would like to thank you for the paragraph template, it was very useful for reformulating the whole discussion.**

It is really beyond my role to rewrite the rest of the text from lines 405-466. This needs however a complete re-write following what I have written above. I have not re-written pages 23-25 lines 426-486 – need careful re-write to bring out the main points. However – a brief look

**Response: We rewrote the paragraphs carefully and thoughtfully.**

Line 425 – delete the entire line as this does not make sense.

**Response: The alteration has been made.**

Line 437 – more issues with past tense – this should be “were” delete “are”

**Response: The alteration has been made.**

5. Conclusion

Lines 481-486 Re write from lines 481-487.

**Response: We have rewritten the second paragraph of the conclusion to make it more objective.**

Final comments to authors - please review this manuscript in detail - and ensure your efforts to date can be used to bring this to a positive conclusion.

**Response: Thank you for the opportunity to review the manuscript once again.**
