# Supplementary material for: Fatigue and resilience in Master’s and PhD students in the Covid-19 pandemic in Brazil: A cross-sectional study
Source: PLoS One. 2023 Dec 1;18(12):e0295218. doi: 10.1371/journal.pone.0295218 (PMC10691712; doi:10.1371/journal.pone.0295218)
Supplement: Attachment — Submitted filename: RESPONSE TO REVIEWERS.docx [file pone.0295218.s010.docx]

**RESPONSE TO REVIEWERS**

**São Paulo, November 16, 2023.**

Dear Professor Pauline M. Ross:

We would like to thank you for the comments on our manuscript. We have made the proposed changes, and below we clarify the suggestions in red point by point.

Yours sincerely,

_________________________________________________________________

Ph.D. candidate at the Nursing School of University of São Paulo. Rua Doutor

Enéas de Carvalho Aguiar, 419, CEP – 05403-000, Cerqueira Cesar, São Paulo, SP,

Brazil. Telephone: 55 11 9 88481679. Corresponding author: izabel.chagas@usp.br

**Academic Editor**

Thank you for submitting your manuscript to PLOS ONE. After careful consideration, we feel that it has merit but does not fully meet PLOS ONE’s publication criteria as it currently stands. Therefore, we invite you to submit a revised version of the manuscript that addresses the points raised during the review process.

In general this manuscript has improved and it is very close to acceptance for publication. I would still like to have the authors seek further advice on sentence structure from a native English speaker. I understand this may have already been done – but it could be repeated. The discussion reads better than the introduction – but the purpose of the introduction is to engage the reader – and more could be done here.

**Response: We have changed the logic and tried to involve the reader in the new version of the introduction.**

Specific areas to fix.

Write the abstract as a coherent 300 word statement without subheadings. Also in the conclusion replace intensify with design – lines 61

**Response: The alteration has been made.**

Add the aims and research question at the end of the introduction – lines 134 – so that the reader understands the purpose of the study clearly.

**Response: We added the questions associated with the objectives that were already described at the end of the introduction.**

Line 175 What is meant here by “horizontal line” – do you mean the scale goes from 0-10? I am not sure how measurements in centimetre are involved? The one I researched on line is a scale from 0-10 – remove reference to cm.

**Response: The alteration has been made.**

Line 312 What is income five minimum wages above? Perhaps you mean “Having an income which is at least five times greater than the minimum wage is correlated with greater resilience” Please use this sentence and replace if correct.

**Response: The alteration has been made.**

Line 314 If these were Master’s students – then why are they retired – what proportion of masters students were retired – is this an important finding? Perhaps reconsider.

**Response: We have modified the text. This paragraph shows that students who have a source of income from retirement are more resilient and this is important data, as it was a statistically significant variable. Of the 10 students whose source of income comes from retirement, 5 are master's students (50%) and have an average age of 60.**

Discussion –

Should mainly be written in the past tense – see line 404 – replace “is” with “was” and check throughout.

**Response: The alteration has been made.**

Line 424 Commence with “This study found” and delete “It was identified”

**Response: The alteration has been made.**

Line 437 Replace “are” with “were” – past tense again.

**Response: The alteration has been made.**
